# A Semi‐Quantitative Yeast Complementation Platform for Characterizing Urea and Ammonia Transport by Membrane Channels

**DOI:** 10.1002/cpz1.70336

**Published:** 2026-03-06

**Authors:** Anna Stoib, Sahar Shojaei, Christine Siligan, Andreas Horner

**Affiliations:** ^1^ Johannes Kepler University Linz, Institute of Biophysics Linz Austria; ^2^ These authors contributed equally to the work

**Keywords:** ammonia transport, membrane channel permeability, semi‐quantitative permeability screening, urea transport, yeast complementation assays

## Abstract

Yeast complementation assays provide a robust *in vivo* platform for characterizing the permeability and pH gating of transmembrane channels. This article details a liquid culture approach to quantify urea and ammonia transport using *Saccharomyces cerevisiae* deletion strains. Functional complementation, evidenced by cell growth in selective medium with urea or ammonia as the sole nitrogen source, directly reports on channel activity, generating solute‐specific permeability and pH‐dependency profiles. We present step‐by‐step procedures using the bacterial urea channel *Hp*UreI of *Helicobacter pylori*, including two variants (A57C and L134C) for urea permeability and *Hp*UreI, *Hp*UreI E177Q, and human *h*AQP8 for ammonia transport. By monitoring growth across a pH range, this method enables semi‐quantitative comparison of channel function. The assay is cost effective, scalable to high‐throughput formats, and adaptable for studying diverse solutes, protein homologs, or mutants. It also serves as an efficient pre‐screening tool for affinity tag placement before *in vitro* characterization. Unlike *in vitro* reconstitution, this approach preserves native protein‐lipid interactions and avoids purification artifacts, allowing direct comparison to wild‐type proteins. Though less quantitatively precise than *in vitro* methods, it offers higher throughput and solute flexibility compared to oocyte expression systems. © 2026 The Author(s). *Current Protocols* published by Wiley Periodicals LLC.

**Basic Protocol**: Quantifying urea permeability and pH gating using a yeast complementation growth assay

**Alternate Protocol**: Adapting the yeast complementation assay to assess ammonia permeability and pH dependency

## INTRODUCTION

Membrane channels are essential for the selective transport of solutes across biological membranes, enabling critical physiological processes and environmental adaptation. These proteins facilitate the passive diffusion of molecules such as urea, ammonia, glycerol, and water, and their dysregulation is implicated in diseases and microbial survival. For example, the pH‐gated urea channel UreI in *Helicobacter pylori* is vital for survival in acidic environments (Stoib, Shojaei, Siligan, et al., 2025), and aquaporins regulate osmotic balance across kingdoms (Anders Blomberg, 2022; Goessweiner‐Mohr et al., 2022). Characterizing the permeability and gating mechanisms of these channels is crucial for understanding their functional roles and potential as therapeutic targets.

### Methods for Measuring Neutral Solute Permeability

Approaches to studying solute permeability include *in silico*, *in vitro*, and *in vivo* methods. Computational simulations provide not only predictions of permeability and gating but also mechanistic insights into channel dynamics, revealing conformational changes, solute interactions, and gating transitions at atomic resolution (McNulty et al., 2013). When validated experimentally (e.g., when molecular dynamics simulations align with functional data), *in silico* approaches become a powerful tool for probing structure‐function relationships (Goessweiner‐Mohr et al., 2022; Pluhackova et al., 2022; Siligan et al., 2025; Wachlmayr et al., 2023).

*In vitro* methods, such as stopped‐flow spectroscopy with proteoliposomes (Hannesschlager et al., 2018; Horner et al., 2015; Wachlmayr et al., 2021) or micro‐aspiration of giant unilamellar vesicles (GUVs; Boytsov et al., 2020), yield high‐resolution kinetic data. However, these techniques are experimentally demanding, requiring protein purification, reconstitution into model membranes, and complex data processing to extract accurate permeability values. Quantitative measurements often necessitate additional steps, such as channel density estimation (for example, via fluorescence correlation spectroscopy; Wachlmayr et al., 2023), to determine single‐channel permeability. Though precise, *in vitro* assays are time‐consuming, cost‐intensive, and technically specialized, with limitations like protein misfolding or loss of native interactions during purification and reconstitution.

*In vivo* systems, such as *Xenopus laevis* oocytes or *Saccharomyces cerevisiae*, enable the study of solute transport in a native physiological context, preserving cellular components like interacting proteins, lipids, and cofactors. Crucially, *in vivo* systems bypass the need for protein purification, which can otherwise alter protein function. However, they are inherently less defined than *in vitro* methods because of the complexity of living cells.

### Yeast as a Model Organism for Studying Membrane Channel Permeability

*Saccharomyces cerevisiae* is a powerful and versatile model for functional studies of membrane channels. Its conserved protein expression machinery, cost‐effectiveness, and simplicity relative to mammalian cell culture make it an ideal system for heterologous expression (Frommer & Ninnemann, 1995; Mohammadi et al., 2015). Yeast supports the overexpression of membrane proteins with minimal host toxicity and shares membrane properties with plant cells, making it suitable for studying plant proteins as well (Bjorkskov et al., 2017). Its ability to grow on solid or liquid media (Simon & Bedalov, 2004) under diverse conditions, including acidic environments, further enhances its utility (Leitão et al., 2012; Simon & Bedalov, 2004).

**Figure 1:**
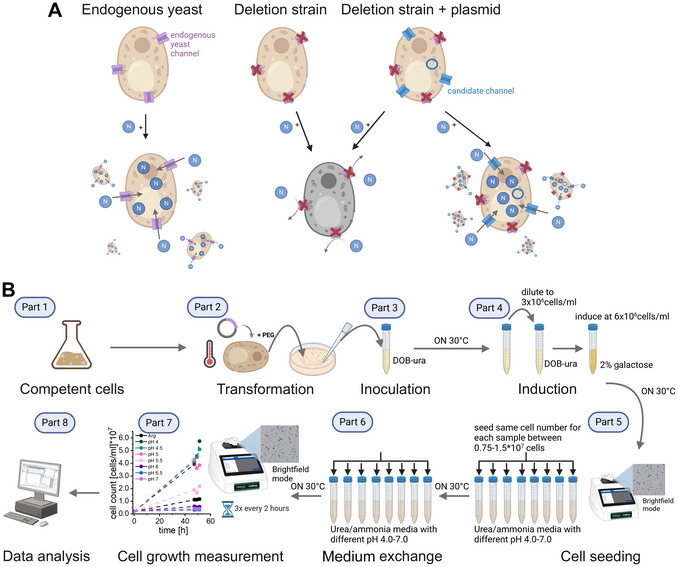
Nitrogen complementation assay. (**A**) Principle: In wild‐type yeast, endogenous channels (DUR3 for urea, MEP1‐3 for ammonia) allow survival when urea or ammonia is the sole nitrogen source. In deletion strains lacking these channels, urea/ammonia can only enter via passive membrane diffusion, limiting nitrogen uptake and causing reduced cell growth. When the deletion strain is transformed with a candidate urea or ammonia channel, its functionality and permeability can be inferred from cell growth: higher permeability results in better growth, as the channel complements the deletion. (**B**) Workflow: Single colonies of transformed constructs are inoculated, and protein expression is induced by switching to galactose‐containing medium for 24 hr. An equal number of cells is then seeded into urea or ammonia media (pH 4.0‐7.0) and arginine control medium. After 24 hr, the media are refreshed. The next day, cell concentrations are measured in duplicate every 2 hr over a 6‐hr period. Growth curves are plotted and data are analyzed further.

The fully sequenced genome of *S. cerevisiae* (Goffeau et al., 1996) and its well‐characterized deletion strain libraries (Baudin et al., 1993) enable precise functional analyses. Yeast complementation assays exploit these deletion strains to test whether exogenous proteins can restore function under selective conditions (Fig. 1A). In these assays, strains lacking a specific gene are transformed with plasmids encoding candidate proteins (Giaever & Nislow, 2014; Groszmann et al., 2023; Mohammadi et al., 2015; Stoib, Shojaei, Siligan, et al., 2025). Growth or survival under conditions requiring the deleted gene's function confirms functional complementation, demonstrating that the candidate protein can substitute for the missing activity.

For urea permeability assays, candidate proteins are heterologously expressed in a yeast deletion strain lacking the endogenous urea channel. When cultured in medium where urea is the sole nitrogen source, strains expressing channels that do not transport urea exhibit reduced growth or growth arrest, as nitrogen is essential for amino acid and nucleotide biosynthesis. Thus, monitoring cell growth provides a semi‐quantitative readout of urea permeability. The same principle applies when ammonia is used as the nitrogen source.

### Applications of Yeast Complementation Assays

Yeast complementation assays are versatile tools for characterizing the permeability of membrane channels to a broad range of solutes, including urea (Klebl et al., 2003; Liu et al., 2003; Wang et al., 2012; Zanin et al., 2014), ammonia (Ariz et al., 2018; Beitz et al., 2006; Jahn et al., 2004; Kirscht et al., 2016; Loque et al., 2005; Williamson et al., 2020; Wu et al., 2008), glycerol (Ferreira et al., 2005; Gotfryd et al., 2018; Karlgren et al., 2005; Pettersson et al., 2006), hydrogen peroxide (Bienert et al., 2011; Bienert et al., 2007; Mao & Sun, 2015), metalloids (Bienert et al., 2008; Fitzpatrick & Reid, 2009; Karlgren et al., 2005; Kumar et al., 2014; Mosa et al., 2016; Sabir et al., 2020), and water (Leitão et al., 2012; Pettersson et al., 2006; Soveral et al., 2011; Tanghe et al., 2004; Tanghe et al., 2002). For toxic solutes such as metalloids, the assay relies on toxicity or sensitivity measurements, with reduced growth indicating higher permeability. The Strategic Planning section provides tips for adapting the Basic Protocol to solutes other than urea and ammonia.

These assays are widely used to compare wild‐type proteins, homologs, or variants and to identify critical amino acids, structural features, or affinity tags that influence channel function (Beitz et al., 2006; Kirscht et al., 2016; Stoib, Shojaei, Siligan, et al., 2025; Stoib, Shojaei, Fischer, et al., 2025; Williamson et al., 2020). They can also assign functions to genes by testing candidate sequences or organismal ORF libraries (Jahn et al., 2004; Liu et al., 2003; Liu, Ludewig, Gassert, et al., 2003; Marini et al., 2000; Zanin et al., 2014). Additionally, yeast complementation assays are valuable for studying drug or inhibitor interactions (Bertl & Kaldenhoff, 2007; Bienert et al., 2007; To et al., 2015), facilitating the identification of potential therapeutics.

Yeast complementation assays can be performed in solid‐medium spotting formats for quick, qualitative assessments (Ferreira et al., 2005; Loque et al., 2005; Marini et al., 2000; Sabir et al., 2020; Sabir et al., 2014; Williamson et al., 2020) or in liquid‐culture formats for semi‐quantitative growth measurements using spectrophotometry or cell counting (Ferreira et al., 2005; Karlgren et al., 2005; Sabir et al., 2020; Stoib, Shojaei, Siligan, et al., 2025). Liquid assays provide a uniform nutrient supply and, when adapted to 96‐well plate formats, enable higher throughput for efficient screening of multiple conditions or compounds (A. Blomberg, 2011; Hung et al., 2018; Karlgren et al., 2005).

### Advantages and Limitations

Yeast complementation assays offer a cost‐effective, time‐efficient, and universal platform for functional testing of proteins and solutes. They preserve native protein‐lipid interactions and avoid purification artifacts, unlike *in vitro* reconstitution. However, they provide semi‐quantitative rather than absolute permeability values and are less precise than *in vitro* methods because of the complexity of living cells.

### Type of Data Obtained

This protocol generates pH‐dependency profiles for urea and ammonia permeability, revealing solute‐specific transport and gating behavior. Growth data, normalized to arginine controls, are transformed into graphs that illustrate the pH‐dependent activity of candidate membrane channels.

### Description of Protocols

This article provides a step‐by‐step guide for performing yeast complementation assays to study urea and ammonia permeability. The Basic Protocol details a liquid‐culture assay to characterize urea permeability using *H. pylori* UreI (*Hp*UreI) and two point mutations (A57C and N‐tag+L134C). The Alternate Protocol adapts this approach for ammonia permeability using *Hp*UreI, a pore‐lining mutant E177Q, and human aquaporin 8 (*h*AQP8). The principle and most steps are identical for both assays, with differences for ammonia testing outlined in the Alternate Protocol. The choice of protocol depends on the solute permeability being characterized.

*NOTE*: Yeast cultures must be handled under sterile conditions to prevent contamination, particularly from bacteria such as *Escherichia coli*. Always work near a Bunsen burner or in a laminar‐airflow hood, and keep yeast‐related activities physically separated from other microbial work. Clean all surfaces and equipment with 70% ethanol before and after use.

*NOTE*: Regularly inspect cultures and media for contamination, either visually or using a cell counter. Contaminated samples, media, or waste must be autoclaved immediately. Prepared media can be stored refrigerated for several weeks, but should be checked for contamination before use. Discard all cultures and liquid waste by autoclaving.

## STRATEGIC PLANNING

### General

The yeast complementation assay (parts 3‐7) must be performed over five consecutive days, with no pause points available. In our lab, we typically test four constructs across eight conditions (32 samples total) in parallel. It is essential to include a negative control and, whenever possible, a positive control in each assay. To optimize throughput, three independent assays can be initiated on consecutive days, enabling the completion of 96 samples within 7 days.

### Adaptation for Other Solutes

Yeast complementation assays are versatile tools for testing and characterizing a wide range of solutes and proteins. To adapt the protocol for urea and ammonia to other solutes or proteins, several key considerations for experimental design and controls must be addressed.

First, determine whether the assay will be conducted as a growth or survival assay. If the solute is essential for cell growth under the assay conditions, perform a growth assay, where improved solute permeability by the tested protein results in enhanced cell growth. If the solute is toxic, use a survival assay, tracking cell viability to assess detoxification or solute permeability. For example, water permeability can be examined by using a freeze‐survival assay (Stoib, Shojaei, Fischer, et al., 2025). In this assay, water‐permeable channels (e.g., aquaporins) enhance cell survival by reducing osmotic stress during freezing, as fewer ice crystals form inside the cells (Tanghe et al., 2002, 2004). Cell viability can be monitored using FDA (live cells, green fluorescence) and PI (dead cells, red fluorescence) (Stoib, Shojaei, Fischer, et al., 2025).

Next, selecting the appropriate yeast deletion strain is critical. Identify the proteins involved in the solute's metabolism and understand how their knockout affects yeast physiology. Consider whether the solute is essential or toxic, its metabolic pathways, and its uptake or excretion mechanisms. The Saccharomyces Genome Database (SGD) is an invaluable resource for exploring gene functions and metabolic pathways. For the freeze‐survival assay, yeast lacking endogenous water channels (strain Y20000) is used (Stoib, Shojaei, Fischer, et al., 2025).

Based on the solute's metabolic pathway, choose a control solute that is part of the same pathway but metabolized independently of the tested solute. For example, arginine is used as a control solute in urea and ammonia assays, whereas unstressed conditions can serve as controls in survival assays. These controls help normalize solute permeability measurements for better comparability across assays.

Protein expression is another critical factor. Codon‐optimize the DNA sequence for yeast to enhance expression efficiency, and use an inducible promoter, such as the *GAL1* promoter, for controlled expression. Ensure that the vector's auxotrophic selection marker matches the deletion strain by testing growth on YPD plates and selection plates—only transformed yeast should grow on selection plates.

Proper controls are essential for reliable results. Include a negative control (e.g., empty vector) in every experiment to confirm that observed effects are due to the tested protein and not endogenous yeast factors. If possible, include a positive control, such as the wild‐type protein for point mutation studies or a known solute channel when testing new permeabilities.

Finally, optimize assay conditions for the solute and protein of interest. If the channel is pH–gated, test media spanning the full pH range of gating. Screen solute concentration, incubation time, and other conditions to identify optimal settings. Testing a broad range of solute concentrations and incubation times ensures significant and reproducible effects compared to the negative control, accounting for passive diffusion or endogenous yeast activity.

## QUANTIFYING UREA PERMEABILITY AND pH GATING USING A YEAST COMPLEMENTATION GROWTH ASSAY

This protocol describes a step‐by‐step procedure for quantifying urea permeability and pH‐gating of membrane channels using a yeast complementation growth assay. The method involves culturing a transformed yeast deletion strain under selective conditions, exposing cells to urea stress across a range of pH levels, and monitoring growth using a cell counter. Yeast cells are identified in bright‐field mode based on their morphology and size, enabling precise quantification. After data analysis, the membrane channel is semi‐quantitatively characterized for its urea permeability and pH‐dependent gating behavior. An overview of the workflow is presented in Figure 1B.

### Materials

*Saccharomyces cerevisiae* deletion strain YNVW1 (Δ*dur3*, Δ*ura3*; complete genotype in Table 1)
Yeast Transformation Kit (Sigma Aldrich, cat. no. YEAST1)
YPD medium (see recipe)
Milli‐Q‐purified water, sterile
Glycerol
Candidate protein *Hp*UreI with point mutations A57C and L134C in pYES2 vector under control of the *GAL1* promoter
Empty vector pYES2, as negative control
100% (absolute) ethanol
DOB‐ura plates (see recipe)
DOB‐ura medium (see recipe)
Galactose medium (see recipe)
Urea pH media at different pHs (4.0‐7.0; see recipe)
Control medium, pH 6.0 (see recipe)

Incubator
10‐, 100‐, and 1000‐µl pipets
Sterile work bench or Bunsen burner
Centrifuge for 15‐ and 50‐ml tubes
100‐ and 500‐ml Erlenmeyer flasks
Spectrometer (Eppendorf)
Thermomixer, compact (Eppendorf)
Cell counter (e.g., Denovix CellDrop FL)
Heating block, 42°C
Bright‐field application cell counter
Microwave
pH meter (Mettler‐Toledo)
Excel (Microsoft)
Origin2022 pro

**Table 1:** Yeast Strains and Proteins Tested in Growth Complementation Assays

Solute tested	Common name	Background	Genotype	Proteins tested in pYES2 vector
Urea	YNVW1 Δ*dur3*	∑23346c	*Mata*, Δ*dur3*, Δ*ura3*	*Hp*UreIWT, *Hp*UreI A57C, *Hp*UreI L134C
Ammonia	Sc18‐Δ*mep1‐3*	31019b	*MATa*, Δ*ura3*, Δ*mep1*, *mep2*Δ::*LEU2*, *mep3*Δ:*:kanMX2*	*Hp*UreI WT, *Hp*UreI E177Q, *h*AQP8

#### Part 1: Preparation of competent cells (2 days)

This section describes the preparation of chemically competent *S. cerevisiae* cells using the Yeast Transformation Kit 1 (Sigma Aldrich) according to the manufacturer's instructions.

1 Inoculate a single colony or scrape cells from a glycerol stock of the YNVW1 strain into 20 ml YPD medium in an Erlenmeyer flask. Incubate overnight at 30°C with shaking at 200‐220 rpm.
*Use a flask with two‐thirds* to one‐half air space to ensure proper aeration. Because the deletion strain lacks a selection marker, use full YPD medium, which increases the risk of contamination. Work under sterile conditions in a laminar‐flow hood or near an open flame. To minimize bacterial contamination, add 100 µg/ml ampicillin to the medium.
2 The next day, measure the OD_600_ of the overnight culture using a spectrometer. Dilute the culture to OD_600_ = 0.3 in 100 ml fresh YPD medium and incubate at 30°C with shaking until the culture reaches OD_600_ = 0.7‐0.8 (exponential phase).
  Harvesting cells at this OD ensures high cell density and optimal competency while maintaining exponential growth.
3 Pellet the cells by centrifugation for 5 min at 3000 × *g*, room temperature, discard the supernatant, and wash the pellet with sterile Milli‐Q water. Resuspend the cells in transformation buffer (provided in the kit).
  Handle cells gently after adding transformation buffer. Avoid vigorous mixing—use slow pipetting or gentle vortexing to resuspend.
4 Aliquot 50 µl of the competent cell suspension into 1.5‐ml microcentrifuge tubes.
5 For long‐term storage at −80°C, supplement the cell suspension with 15% glycerol before aliquoting. Wrap the aliquots in two layers of paper towels before freezing.
  Freshly prepared competent cells yield the highest transformation efficiency. However, frozen aliquots remain viable for 6‐8 months at –80°C, offering flexibility in experimental planning. Wrapping in paper towels reduces the freeze rate, improving cell viability upon thawing.
  The frozen competent cell aliquots from step 5 can be stored at –80°C for up to 6‐8 months. Note that transformation efficiency may decrease with prolonged storage.

#### Part 2: Transformation of plasmids (∼1.5 hr preparation + 2‐3 days incubation for four constructs)

This section describes the transformation of candidate plasmids into YNVW1 competent cells using the Yeast Transformation Kit 1 (Sigma Aldrich), with minor modifications to the manufacturer's protocol. Perform this step at least 2‐3 days before the assay to allow transformed colonies to grow on selection plates. For best comparability, transform all candidate plasmids simultaneously using the same batch of competent cells.

6 Add 10 µl of 10 mg/ml single‐stranded carrier DNA (provided in the kit) to 50 µl YNVW1 competent cells in a 1.5‐ml microcentrifuge tube.
  Carrier DNA enhances transformation efficiency by protecting plasmid DNA from degradation.
7 Add 0.1 µg of candidate plasmid DNA (e.g., empty vector pYES2, *Hp*UreI WT, or pYES2 carrying point mutations) to the competent cells.
  Use the same amount of DNA for all constructs to ensure consistency.
8 Add 600 µl plate buffer (provided in the kit) to each transformation reaction. Mix by gentle vortexing.
9 Incubate the samples for 30 min at 30°C with shaking at 200‐220 rpm.
  This step allows the DNA to interact with the competent cells.
10 Heat‐shock the cells for 15 min at 42°C.
  Heat shock induces DNA uptake into the yeast cells.
11 Pellet the cells by centrifugation for 30 sec at 10,000 × g, room temperature. Discard the supernatant.
12 Resuspend the pellet in 300 µl sterile Milli‐Q‐purified water by pipetting up and down gently.
13 Plate 100‐150 µl of the transformed cell suspension onto DOB‐ura selection plates using a sterile spreader (sterilize the spreader by dipping it in absolute ethanol and flaming it briefly). Spread the cells in circular motions.
  Ensure that the spreader is not too hot when spreading cells. Cool it by touching the agar surface after the flame disappears (blue flame indicates residual ethanol).
14 Seal the plates and incubate them upside down at 30°C for 2‐3 days.
  Inverting the plates prevents condensation from dripping onto the colonies.
  Transformed colonies on the selection plates can be stored at 4°C for up to 1 month. Healthy colonies should appear silky white, round, and slightly domed. Avoid using colonies with irregular shapes or discoloration.
  For details concerning troubleshooting of this step, see the Troubleshooting section of the Commentary, below.

#### Part 3: Day 0—inoculation of cell cultures (∼10 min)

This section describes the inoculation of liquid cultures from transformed yeast colonies.

15 Use a sterile yellow (200‐µl) pipet tip to scoop a single colony of transformed cells from the selection plate. Eject the tip directly into 5 ml DOB‐ura medium in a 15‐ml sterile blue‐cap tube to transfer the colony.
  Ensure that the colony is well isolated to avoid contamination or mixing of constructs.
16 Incubate the cultures overnight (16‐18 hr) at 30°C with shaking at 180 rpm.
  Loosen the cap of the blue‐cap tube to allow aeration during incubation.
  The overnight cultures will be used for the assay on Day 1. Ensure that the cultures are visibly turbid before proceeding.

#### Part 4: Day 1—induction of protein expression (∼5‐6 hr)

This section describes the induction of protein expression in yeast cultures using galactose medium.

17 Measure the cell concentration of the overnight (ON) cultures using a cell counter. Take a 20‐µl aliquot of each culture and dilute it 10‐fold with sterile Milli‐Q water.
  Mix thoroughly before taking the aliquot and the measurement.
18 Measure 10 µl of the diluted sample twice in bright‐field mode using the following settings:
  Small cell mode: Enable
  Diameter: 2‐15 µm
  Roundness: 30
  Chamber height: 100 µm
  Dilution factor: 10
  Settling time: 40 sec
    The majority of cells should be circled in blue, indicating successful detection. A detailed explanation of the measurement procedure can be found in the Critical Parameters section of the Commentary.
    For details concerning troubleshooting of this step, see Troubleshooting, below.
19 Note the cell concentration for each sample and calculate the average.
20 Dilute the cultures to 2‐3 × 10^6^ cells/ml in 5 ml fresh DOB‐ura medium. Incubate at 30°C with shaking at 180 rpm.
21 Allow the cultures to grow until they reach a concentration of 6 × 10^6^ cells/ml, which typically takes 2‐3 hr.
22 Harvest the cells by centrifugation for 2.5 min at 7400 × *g*, room temperature. Discard the supernatant.
23 Wash the cell pellets with 5 ml sterile Milli‐Q water. Centrifuge again for 2.5 min at 7400 × *g*, room temperature, and discard the supernatant.
24 Resuspend the pellets in 10 ml galactose medium to induce protein expression.
  Galactose induces the GAL1 promoter in the pYES2 vector, driving expression of the candidate protein.
25 Incubate the cultures overnight (16‐18 hr) at 30°C with shaking at 180 rpm.
  Loosen the cap of the blue‐cap tube to ensure proper aeration during incubation.
  The cultures will now be ready for the urea permeability assay on Day 2.

#### Part 5: Day 2—seeding in urea pH media (∼4 hr)

This section describes the preparation of yeast cultures for the urea permeability assay under different pH conditions.

26 Measure the cell concentration of the induced cell cultures as described in steps 17‐18.
27 Calculate the volume required to seed 2.5 × 10^7^ cells for each construct and each of the eight conditions (seven urea pH media with pHs ranging from pH 4 to 7 plus one control medium). If the calculated seeding volume exceeds 1.25 ml, adjust the seeding concentration using Equation 2 (see Critical Parameters, below).
  It is critical that all conditions and constructs start with the same cell concentration to ensure comparability of growth rates. The seeding cell count can range from 0.75 × 10^7^ to 2.5 × 10^7^ cells.
  For details concerning troubleshooting of this step, see Troubleshooting, below.
28 Aliquot the calculated seeding volume into eight separate 15‐ml blue‐cap tubes, one for each condition.
29 Pellet the cells by centrifugation for 2.5 min at 7400 × *g*, room temperature. Discard the supernatant.
30 Resuspend each pellet in 5 ml of the appropriate urea pH medium (pH 4‐7) or control medium (pH 6.0).
31 Incubate the cultures overnight at 30°C with shaking at 180 rpm.
  Loosen the caps of the blue‐cap tubes to allow proper aeration during incubation.
  The cultures are now prepared for growth analysis on Day 4, after the medium exchange on Day 3.

#### Part 6: Day 3—medium exchange (∼1 hr for four samples)

This section describes refreshing the medium to maintain logarithmic growth and prevent cells from entering the stationary phase.

32 Pellet the cells by centrifugation for 2.5 min at 7400 × *g*, room temperature. Discard the supernatant.
  Ensure that the pellet is undisturbed to prevent cell loss and thus cell concentration changes due to medium exchange.
33 Resuspend each pellet in 5 ml fresh urea pH medium (pH 4‐7) or control medium.
  Medium exchange ensures that cells remain in the logarithmic growth phase and avoids nutrient depletion.
34 Incubate the cultures overnight at 30°C with shaking at 180 rpm.
  Loosen the caps of the blue‐cap tubes to allow proper aeration during incubation.
  The cultures will now be ready for cell growth tracking on Day 4.

#### Part 7: Day 4—measuring cell growth (∼7‐8 hr for four samples)

This section describes how to track cell growth over time to generate growth curves for each condition.

35 Take aliquots from each sample and condition, dilute them 10‐fold with Milli‐Q water, and measure the cell concentration in duplicate as described in steps 17 and 18.
  Record the exact time of each measurement to calculate incubation intervals for the growth curve.
36 Document the cell concentration of each sample in an Excel file.
37 Calculate the average cell concentration for each time point.
38 Repeat steps 35‐37 every 2‐2.5 hr, aiming to collect three or four time points for each sample.
39 Export bright‐field images and cell counting results as .png files, and save the cell counts and settings as .csv files for further analysis.
  The data collected can be stored and analyzed later, in part 8. Ensure that all files are clearly labeled for easy reference.

#### Part 8: Data evaluation—growth assay (45 min)

This section describes how to analyze and visualize the growth assay data to characterize urea permeability and pH‐gating.

**Figure 2:**
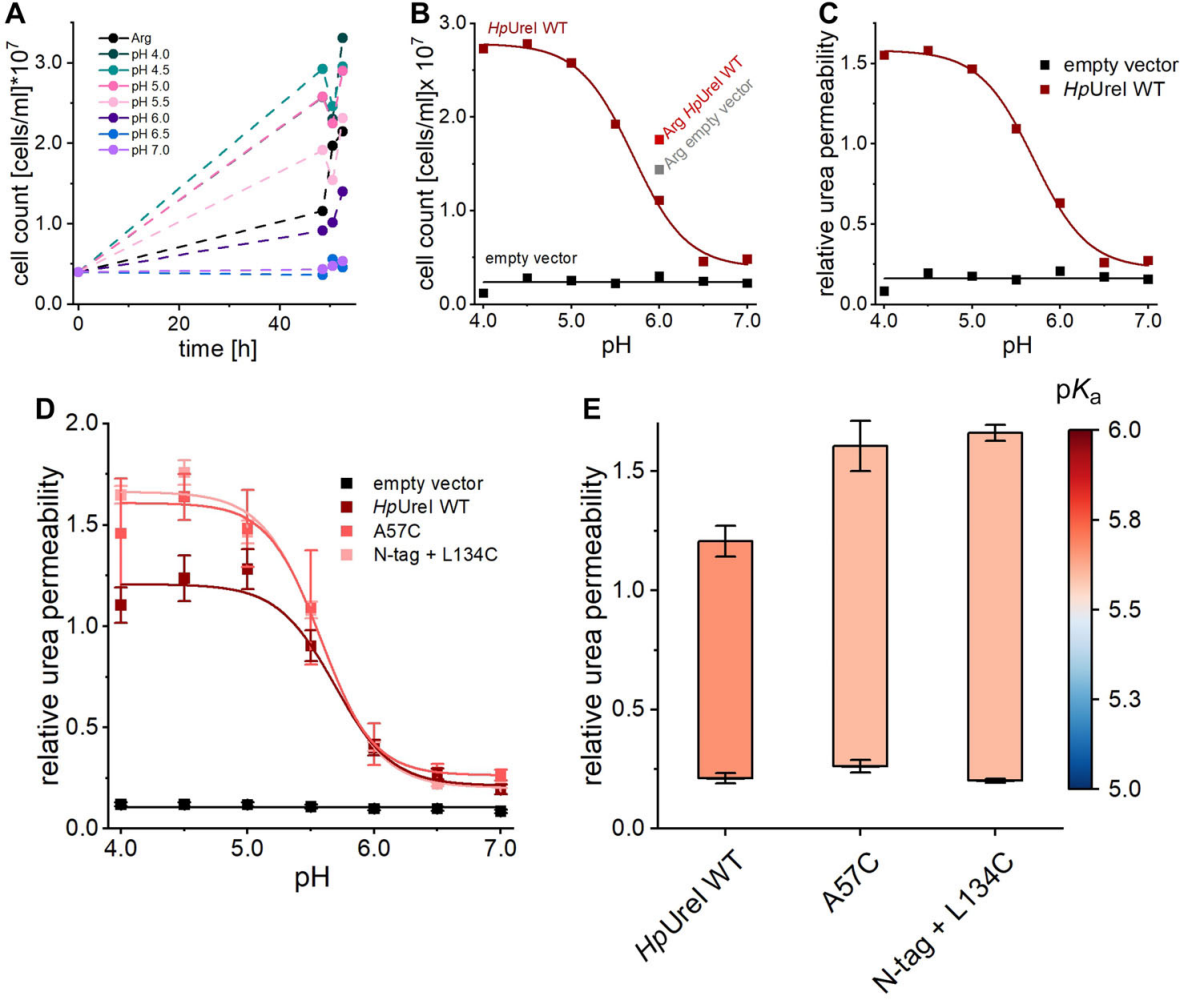
Analysis of pH‐dependent urea permeability in yeast growth assays. (**A**) Growth curves of WT *Hp*UreI showing cell counts on the second day in urea media (pH 4.0‐7.0) and arginine control medium. (**B**) Average cell counts for each pH condition. (**C**) Data normalized to arginine control to determine relative urea permeability, plotted against medium pH to illustrate pH‐dependent urea permeability. (**D**) Averages of relative urea permeabilities ± SEM from independent assays for empty vector (*n* = 30), WT *Hp*UreI (*n* = 18), A57C (*n* = 3), and N‐tag + L134C (*n* = 5), fitted to a Boltzmann function. (**E**) p*K*_a_ values (color‐coded) and Δurea values extracted from the fits are presented in bar charts.

40 Import data: Import the time point averages into OriginPro 2022 or an equivalent software.
41 Generate growth curves: Plot the time point averages for each sample across different conditions over the incubation time to create growth curves (Fig. 2A).
  Calculate incubation time from the start of incubation in urea pH medium to each aliquot measurement.

42 Calculate day averages: Compute the daily average cell concentration for each condition.
43 Plot unnormalized pH‐gating curves: Create a point diagram of the medium pH versus day averages to generate unnormalized pH‐gating curves (Fig. 2B).
44 Normalize data: Normalize each pH condition's day average to the arginine control medium average for the same sample.
45 Plot normalized pH‐gating curves: Generate a point diagram of the medium pH versus normalized day averages to obtain pH‐gating curves (Fig. 2C).
  For details concerning troubleshooting of this step, see Troubleshooting, below.
46 Average independent assays: To compare different constructs, calculate the mean of at least three independent assays and determine the standard error of the mean (SEM).
  Ensure that the negative control exhibits low normalized urea permeability; if not, exclude the assay from analysis. Optionally, perform a Grubbs outlier test on the normalized day averages and exclude any identified outliers from further analysis.
47 Plot averaged data with SEM: Create a *y*‐error bar point diagram of the normalized averages from independent assays, using SEM as *y*‐error bars (Fig. 2D).
48 Fit pH‐dependent data: Select the pH‐dependent dataset for, e.g., *Hp*UreI.
  a. In the right app panel, click Simple Fit.
  b. Under the Non‐linear header, select Use Existing Function.
  c. Choose Growth/Sigmoidal from the left dropdown menu, and then select the Boltzmann function from the right dropdown menu.
  d. Fit the data using the following function:
(1) $$y=\frac{A_{1}-A_{2}}{1+e^{\left(x-x_{0}\right){/}_{dx}}}+A_{2}$$
  e. Click Fit to apply the function.
  f. If the fit aligns with the data points and lies within the error bars, click Report. If not, click Re‐initialize and attempt the fit again.
49 Extract p*K*_a_ and $\Delta_{\mathrm{urea}}$: A Simple Fit Summary will appear in the data sheet. Extract the p*K*_a_ value (*x*_0_) from the fit. Use *A*_1_ and *A*_2_ as the minimal and maximal relative urea permeability ($\Delta_{\mathrm{urea}}$).
50 Visualize results: Plot the fitted and extracted values for p*K*a and $\Delta_{\mathrm{urea}}$ in a bar chart with their SEM as *y*‐error bars (Fig. 2E).
  If the sigmoidal curve does not fit the experimental data, consider alternative fitting functions.

## ADAPTING THE YEAST COMPLEMENTATION ASSAY TO ASSESS AMMONIA PERMEABILITY AND pH DEPENDENCY

This protocol adapts the yeast complementation assay for ammonia permeability and pH dependency. The procedure is similar to the urea growth assay, with changes limited to the yeast deletion strain and assay media. Below are the key modifications to the affected protocol steps.

### Additional Materials (also see Basic Protocol)

*Saccharomyces cerevisiae* deletion strain Sc18‐Δ*mep1‐3* (Δ*mep‐1‐3*, Δ*ura3*; background: 31019b; full genotype in Table 1)
Vectors encoding candidate proteins: *Hp*UreI, *Hp*UreI E177Q, and *h*AQP8 in pYES2 vector under the control of the *GAL1* promoter
Empty vector pYES2, as negative control
Ammonia pH media at different pHs (4.0‐7.0; see recipe)
Control medium, pH 6.0 (see recipe)

Perform the Basic Protocol with the following modifications to the indicated steps:

#### Part 1: Preparation of competent cells (2 days)

1 Use the Sc18‐Δ*mep1‐3* deletion strain for inoculation and preparation of competent cells.

#### Part 2: Plasmid transformation (∼1.5 hr +3‐4 days of incubation for four constructs)

6 Use Sc18‐Δ*mep1‐3* competent cells to set up the transformation reactions.
7 Add 0.1 µg of the following candidate plasmids to the appropriate reactions: empty vector pYES2, *Hp*UreI WT, *Hp*UreI E177Q, and *h*AQP8.

#### Part 5: Day 2—seeding in ammonia pH medium (∼4 hr)

28 Aliquot the calculated seeding volume into eight separate blue‐cap tubes, one for each condition (seven with ammonia media at pHs from 4.0 to 7.0 plus one with control medium).
30 Resuspend each pellet in 5 ml ammonia pH medium or control medium.

#### Part 6: Day 3—medium exchange (∼1 hr for four samples)

33 Resuspend pellets in 5 ml fresh ammonia pH medium or control medium.

#### Part 8: Data evaluation—growth assay (45 min)

**Figure 3:**
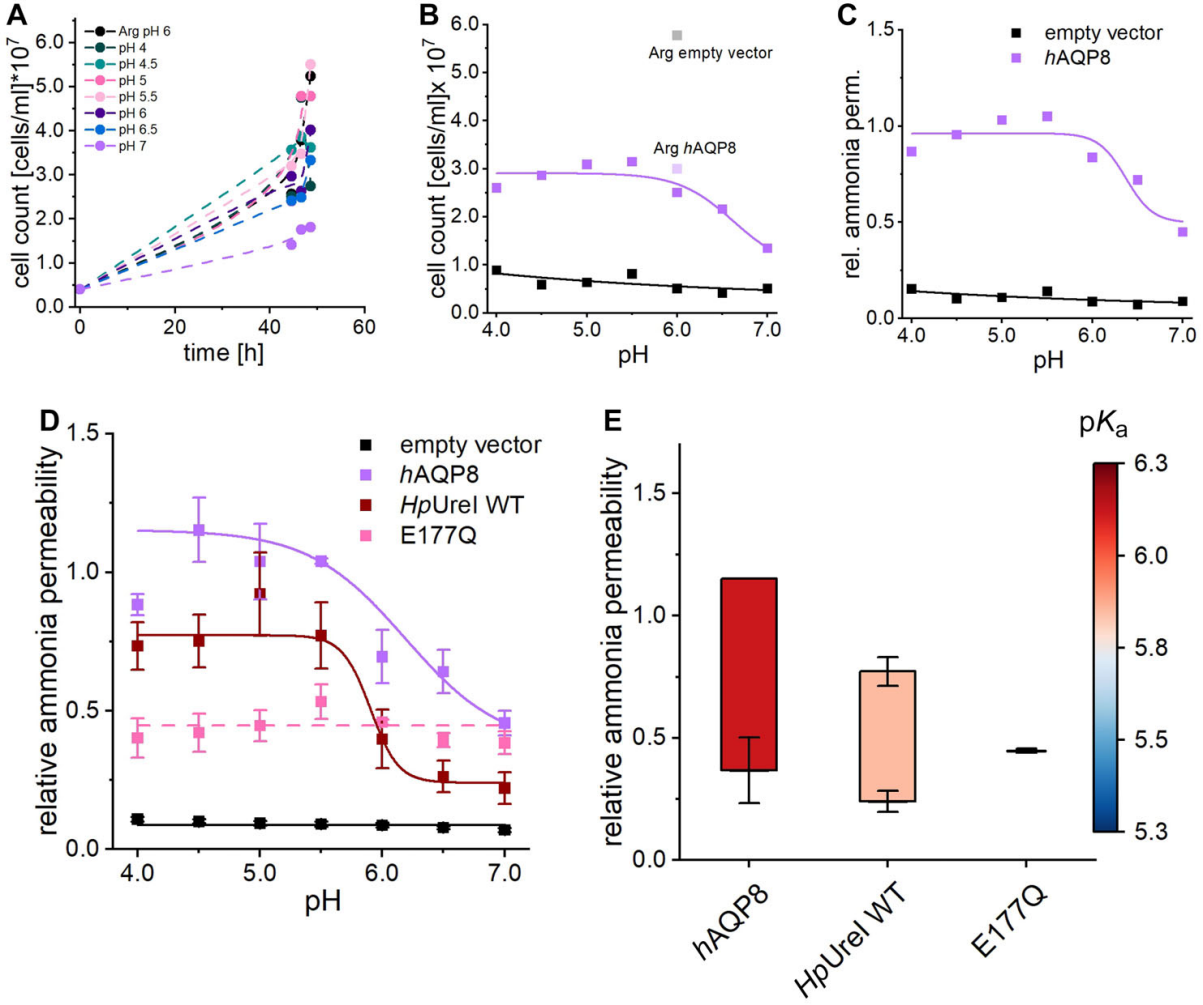
Analysis of pH‐dependent ammonia permeability in yeast growth assays. (**A**) Example growth curves of hAQP8 in ammonia media (pH 4.0‐7.0) and arginine (Arg) control medium. (**B**) Unnormalized pH dependency graph: Day‐averaged cell counts for each pH condition, plotted for *h*AQP8 and the empty vector (negative control). (**C**) Normalized pH dependency: Data normalized to the Arg day average, showing the pH‐dependent ammonia permeability. (**D**) Averaged relative ammonia permeability (± SEM) from independent assays for the empty vector (*n* = 18), *h*AQP8 (*n* = 3), *Hp*UreI (*n* = 7), and E177Q (*n* = 3). (**E**) Extracted p*K*_a_ values (color‐coded) and ammonia permeability amplitudes, summarized in a bar chart.

- Exemplary graphs of the ammonia assays are presented in Figure 3.
- Fit samples to a Boltzmann function and extract p*K*_a_ and $\Delta_{\mathrm{ammonia}}$ values.
- For *Hp*UreI E177Q, where the sigmoidal fit fails, use a linear fit with the slope set to 0. Extract the *y*‐intercept from the fit data and plot $\Delta_{\mathrm{ammonia}}$ as a single point in Figure 3E.

## REAGENTS AND SOLUTIONS

To prepare media, it is advisable to set up stock solutions for some reagents (urea, ammonia, arginine, buffers, and YNB), which can be stored at 4°C for several months. Most media can be sterilized by autoclaving or microwaving. For microwaving, heat the solution to boiling 2‐3 times. Microwaving is a flexible alternative for smaller volumes and is less degrading to ingredients because of the shorter heat exposure. For larger volumes or thorough sterilization, autoclaving is preferred.

*NOTE*: Regularly check cultures and media for contamination, either visually or with a cell counter. Prepared media can be stored in the refrigerator for several weeks, but must be inspected for contamination before use.

*CAUTION*: Use appropriate heat‐resistant gloves and safety equipment when handling hot glassware during microwaving.

### Ammonia pH media/control arginine medium (200 ml)

Dissolve in 200 ml Milli‐Q‐purified water:
20 ml of 10× YNB stock (see recipe; 1× final)
20 ml of 1 M succinate‐Tris buffer (see recipe; 100 mM final)
4 g d‐galactose (2% [w/v] final; Apollo Scientific, cat. no. BIG1724)
Adjust pH to 4.0‐7.0 for ammonia media or pH 6.0 for control arginine medium
Sterilize by autoclaving or by microwaving (bringing to a boil 2‐3 times)
Allow medium to cool
*If autoclaving*: Add galactose after cooling
Add:
4 ml 50 mM arginine stock (see recipe; 1 mM final) for control medium
4 ml 100 mM ammonia stock (see recipe; 2 mM final) for ammonia media
Store up to 1 month at 4°C
  Tip: Prepare a larger volume and split into smaller portions before pH adjustment. Adjust volume based on assay needs (∼10‐15 ml per condition per sample).

### Ammonia stock, 100 mM (50 ml)

Dissolve 0.267 g ammonium chloride (100 mM final; molecular biology grade; Sigma‐Aldrich, cat. no. A9434) in 50 ml Milli‐Q‐purified water. Sterilize by filtration using a 0.2‐µm‐pore‐size filter (Millipore, cat. no. SLGSR33SS; note that autoclaving or heating may degrade ammonia). Store up to ∼2 months at 4°C.

### Arginine stock, 50 mM (50 ml)

Dissolve 0.435 g l‐arginine base (50 mM final; BioChemica, IWT reagents, cat. no. A3675) in 50 ml Milli‐Q‐purified water. Sterilize by filtration using a 0.2‐µm‐pore‐size filter (Millipore, cat. no. SLGSR33SS; note that autoclaving or heating may degrade arginine). Store up to ∼2 months at 4°C.

### DOB‐ura medium and plates (for overnight cultures and selection plates; 200 ml or ∼8 plates)

Dissolve in 200 ml Milli‐Q‐purified water:
5.4 g DOB (MP Biomedicals, cat. no. 4025022)
0.154 g CSM‐ura (MP Biomedicals, cat. no. 4511222)
*For plates*: Add 4 g agar (2% [w/v] final; Sigma‐Aldrich, cat. no. 05040)
Sterilize by autoclaving or by microwaving (bringing to a boil 2‐3 times)

*For plates*: Allow the solution to cool to hand warm (∼50°C) before pouring (∼25 ml per plate). Let plates solidify for 20‐30 min and then store in a sealed plastic bag at 4°C (upside down to prevent drying).

Store medium and plates up to 1 month at 4°C.

### Galactose medium (for induction; 200 ml)

Dissolve in 200 ml Milli‐Q‐purified water:
20 ml 10× YNB stock (see recipe; 1× final)
0.154 g CSM‐ura (MP Biomedicals, cat. no. 4511222)
4 g d‐galactose (if microwaving medium; 2% [w/v] final; Apollo Scientific, cat. no. BIG1724)
Sterilize by autoclaving or by microwaving (bringing to a boil 2‐3 times)
Allow to cool
*If autoclaving*: Add galactose after cooling
Add 8 ml of 50 mM arginine stock (see recipe; 1 mM final)
Store up to 1 month at 4°C

### Succinate‐BisTris‐MOPS buffer, 1 M (100 ml)

Dissolve in 70 ml Milli‐Q‐purified water using a magnetic stirrer:
16.2 g sodium succinate dibasic (≥98%, anhydrous; Sigma‐Aldrich, cat. no. 14160)

20.9 g BisTris (≥99%; Fisher BioReagents, cat. no. BP301)
20.9 g MOPS (molecular biology grade; Fisher BioReagents, cat. no. BP308)
Adjust pH to 5.5 with concentrated HCl or NaOH (may require up to 10 ml concentrated HCl)

Bring to 100 ml with Milli‐Q water
Store up to ∼6 months at 4°C
*CAUTION*: Sodium succinate dibasic is a fine powder that may irritate the airways. Weigh it in a well‐ventilated area or under a fume hood, and always use appropriate PPE (mask/respirator, gloves, safety glasses).
*CAUTION*: Handle concentrated HCl with care. Wear gloves and work in a fume hood, as HCl is corrosive and fuming.

### Succinate‐Tris buffer, 1 M (100 ml)

Dissolve in 70 ml Milli‐Q‐purified water using a magnetic stirrer:
16.2 g sodium succinate dibasic (≥98%, anhydrous; Sigma‐Aldrich, cat. no. 14160)

12.1 g Tris base (molecular biology grade; Fisher BioReagents, cat. no. M27435)
Adjust pH to 5.5 with concentrated HCl or NaOH (may require up to 10 ml concentrated HCl)
Bring to 100 ml final with Milli‐Q water
Store up to ∼6 months at 4°C
*CAUTION*: Sodium succinate dibasic is a fine powder that may irritate the airways. Weigh it in a well‐ventilated area or under a fume hood, and always use appropriate PPE (mask/respirator, gloves, safety glasses).
*CAUTION*: Handle concentrated HCl with care. Wear gloves and work in a fume hood, as HCl is corrosive and fuming.

### Urea pH medium/control arginine medium (200 ml)

Dissolve in 200 ml Milli‐Q‐purified water:
20 ml of 10× YNB stock (see recipe; 1× final)
20 ml of 1 M succinate‐BisTris‐MOPS buffer (see recipe; 100 mM final)
4 g d‐galactose (if microwaving medium; 2% [w/v] final; Apollo Scientific, cat. no. BIG1724)
Adjust pH to 4.0‐7.0 for urea media or pH 6.0 for control arginine medium
Sterilize by autoclaving or by microwaving (bringing to a boil 2‐3 times)
Allow medium to cool
*If autoclaving*: Add galactose after cooling
Add 4 ml of 100 mM urea stock (see recipe; 2 mM final) for urea media or 4 ml of 50 mM arginine stock (see recipe; 1 mM final) for control medium. Store up to 1 month at 4°C.
  Tip: Prepare a larger volume and split into smaller portions before pH adjustment. Adjust volume based on assay needs (∼10‐15 ml per condition and sample).

### Urea stock, 100 mM (50 ml)

Dissolve 0.300 g urea (100 mM final; VWR, cat. no. 0568) in 50 ml Milli‐Q‐purified water. Sterilize by filtration using a 0.2‐µm‐pore‐size filter (Millipore, cat. no. SLGSR33SS; note that autoclaving or heating above 37°C may degrade urea into ammonia). Store up to ∼6 months at 4°C.

### YNB, 10× stock (250 ml)

Dissolve 4.75 g Yeast Nitrogen Base (without amino acids and ammonium sulfate; Formedium, cat. no. CYN0505) in 250 ml Milli‐Q‐purified water. Sterilize by autoclaving or by microwaving (bringing to a boil 2‐3 times). Store up to 6 months at room temperature; once opened, store at 4°C. Dilute 10× for medium preparation.

### YPD medium (full medium, for competent cells, 200 ml)

Dissolve in 200 ml Milli‐Q‐purified water:
2.0 g yeast extract (1% [w/v] final; ultrapure; Thermo Scientific, cat. no. J23547.A1)
4.0 g peptone casein (2% [w/v] final; enzymatic digest; Millipore, cat. no. 82303)
4.0 g d‐glucose monohydrate (2% [w/v] final; molecular biology grade; Roth, cat. no. 68871)
Sterilize by autoclaving or by microwaving (bringing to a boil 2‐3 times). Allow medium to cool before use. Store up to 1 week at 4°C; check for contamination before use, as this is a full medium without selection markers.

## COMMENTARY

### Critical Parameters

#### Competent cells and transformation

To achieve a high yield of transformed yeast cells, it is critical to use freshly prepared competent cells. Avoid overgrowth during preparation (Step 2), as cells must remain in the exponential growth phase. Optimal transformation efficiency is typically achieved when the OD_600_ is between 0.7 and 0.8, ensuring that the cells undergo at least one doubling during this phase.

For long‐term storage of competent cells, add glycerol to 15% (v/v) to the cell suspension and mix thoroughly. Aliquot 50 µl portions to minimize freeze‐thaw cycles, which can significantly reduce cell viability. To improve the viability of frozen aliquots, wrap the tubes in several layers of paper towels to slow the freezing rate. Note that transformation efficiency decreases with prolonged storage.

To mitigate reduced efficiency, the pellet in step 12 can be resuspended in a smaller volume (150‐200 µl) to increase cell density.

For consistency and comparability across experiments, transform all constructs into the same batch of competent cells.

#### Growth assays

To ensure robust overexpression of the target protein, induce protein expression by switching the growth medium to galactose‐containing medium during the mid‐log phase, when the cell density reaches ∼6 × 10^6^ cells/ml. Thoroughly wash the cells to remove residual glucose from the previous DOB‐ura medium, as glucose represses the *GAL1* promoter and can reduce protein expression levels.

To initiate the complementation assay, seed the induced cells into urea/ammonia media at various pH levels, as well as into arginine control medium. To ensure comparability, maintain a consistent starting cell concentration across all constructs and conditions. Measure the cell concentration of the induced cultures using a cell counter (detailed settings are provided in the protocol). The starting concentration is determined based on the lowest cell concentration (*c*_min_) among the constructs.

Eight equal aliquots are required: one for the control medium and seven for the different pH conditions. Given that the cultures are induced in 10 ml volume, a maximum of 1.25 ml (*V*_s_) can be seeded per condition in a final volume of 5 ml (*V*_a_). Calculate the seeding concentration using the following formula:

(2) $$c_{\mathrm{seed}}=\frac{c_{\min}*V_{s}}{V_{a}}$$

The starting concentration should range between 1.5 × 10^6^ and 5 × 10^6^ cells/ml. Higher concentrations may result in overly dense cultures by the third day of the assay, whereas lower concentrations can lead to reduced homogeneity across conditions.

After calculating the seeding volume and concentration, divide the samples between the eight conditions. Centrifuge the cells and resuspend the pellet in the appropriate assay medium to ensure that residual induction medium does not dilute the assay medium or interfere with pH or solute concentrations. Because cell growth over time serves as an indirect measure of urea/ammonia permeability, handle the pellet carefully during medium exchange (steps 29 and 32) to avoid loss or inconsistency.

#### Assay controls and optimization

To validate the assay, it is essential to include two controls: an empty vector as a negative control and arginine as a control medium. The media must be free of any additional nitrogen sources. Use yeast nitrogen base (YNB) without ammonia or amino acid supplements to prevent interference with the assay.

The concentration of urea or ammonia may need optimization based on the specific goals of the assay. The optimal solute concentration should maximize the signal‐to‐background ratio. High solute concentrations can lead to increased passive membrane diffusion, which reduces the assay's sensitivity. Conversely, low concentrations may fail to produce measurable growth differences between active and inactive channels.

**Figure 4:**
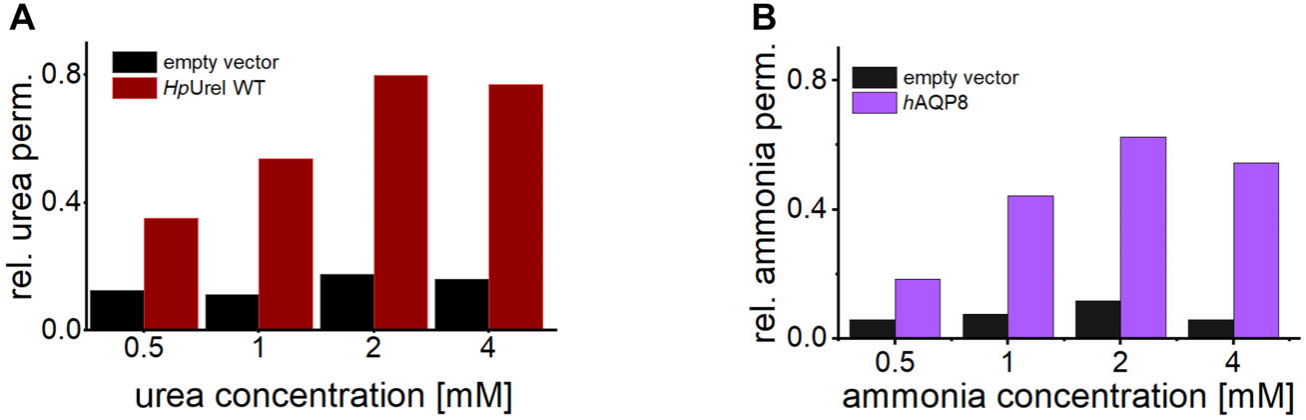
Optimization of nitrogen source concentration. (**A**) Urea and (**B**) ammonia concentration testing for growth assays. To determine the optimal solute concentration for maximal differentiation between background permeability (empty vector) and channel‐mediated permeability, concentrations of 0.5, 1.0, 2.0, and 4.0 mM were tested at pH 4.5. Normalized permeabilities for WT *Hp*UreI (urea) and *h*AQP8 (ammonia) are plotted. For both assays, 2 mM was identified as the optimal concentration.

A concentration screening (1‐10 mM) was conducted using the negative control and the channel to determine the optimal conditions. For both urea and ammonia, a concentration of 2 mM yielded the greatest difference compared to the negative control (Fig. 4). Arginine at 1 mM is used as a control because it metabolizes into two molecules of urea, ensuring equivalent nitrogen levels in the cells.

The buffer in the assay media is formulated to maintain pH stability across the tested range. If the pH range is extended, the buffer composition must be adjusted and tested for potential effects on yeast growth, especially at more alkaline pH levels, as yeast is acidophilic and thrives best in a pH range of 4.0‐7.0.

#### Tracking cell growth

Cell concentrations are measured ∼48 hr after incubation in the assay medium, as this time point typically provides the greatest differences between samples, conditions, and the negative control. Shorter incubation times may result in less pronounced differences, whereas longer incubations can lead to lower cell concentrations and greater variability.

To ensure optimal nutrient availability and prevent cells from entering the stationary phase, the assay medium should be replaced every 24 hr. This step helps maintain consistent growth conditions, ensuring that the measured cell growth accurately reflects urea or ammonia permeability rather than nutrient limitation or stress responses.

#### Cell concentration determination using a cell counter

Accurate determination of cell concentration is critical for calculating relative permeabilities and ensuring robust, reproducible data. To achieve this, follow the following steps carefully.

For sample preparation, thoroughly vortex samples before taking aliquots to ensure homogeneity. Pipet 20 µl of the sample and dilute it with 180 µl of Milli‐Q‐purified water (10× dilution). Vortex the diluted sample again before measurement to ensure even distribution. If the cells appear too dense on the screen, further dilute the sample (e.g., 100×) and adjust the dilution factor in the protocol to calculate the correct cell concentration.

For the measurement procedure using the CellDrop cell counter (DeNovix), begin by checking the measurement chamber. Ensure that the chamber is clean by inspecting the screen image before each measurement—no cells, fibers, or ethanol droplets should be visible. To load the sample, pipet 10 µl of the 10× diluted sample into the cell counter with the arm lowered. Use a white pipet tip to guide the liquid along the groove of the glass plate until it reaches the arm, dispensing the sample slowly. The liquid will be absorbed into the measurement chamber by capillary action and should appear on the screen.

**Figure 5:**
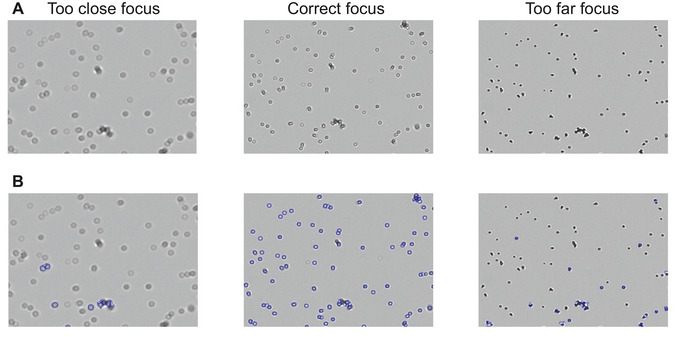
Correct focus setting for cell counting. (**A**) Bright‐field images observed on the cell counter screen during measurement. Middle image: Correct focus—cells display a dark outline and bright interior, enabling accurate detection. Left image: Focus set too close—cells appear faint and blurry, preventing proper recognition. Right image: Focus set too far—cells appear as dark, indistinct dots. (**B**) Result images showing cell detection. Only cells in the middle image are correctly outlined in blue and included in the count. Incorrect focus (left and right) results in failed cell detection, as indicated by the absence of blue outlines.

Allow the cells to settle for 40 sec and then adjust the focus so that cells display a black outline and bright interior (as shown in Fig. 5). Press “Measure” and verify that most cells are circled in blue. Record the cell concentration, and repeat the measurement for each sample twice to ensure accuracy.

### Troubleshooting

Table 2 lists problems that may arise with this procedure along with their possible causes and solutions.

**Table 2:** Troubleshooting Guide for Yeast Complementation Assays

Step	Problem	Possible cause(s)	Solution
14	Very low number of colonies on selection plates	Old competent cells	Prepare fresh competent cells.
		Competent cells with strong cell walls or not in exponential growth phase	Add 10% DMSO in step 8.
		Low cell density of competent cell aliquot	Resuspend pellet in 150 µl Milli‐Q‐purified water in step 12.
18	Majority of cells not blue circled	Wrong focal plane	Adjust focus to visualize cells as dark circles with white interiors (see Fig. 5). Zooming in while focusing may help.
		Unsettled cells	Increase settling time to ensure that cells are in the same focal plane.
		Too many cells	Dilute 100× in step 17.
		Wrong protocol	Switch to the yeast bright‐field protocol.
	Small (moving) cells in background	Contamination	Discard the sample and check for contamination sources (media, water, plates).
	Cell dilution not entering cell counter	Electrostatics	Apply 70% ethanol directly to the glass slides, gently open and close the arm 2‐3 times, and wipe dry with a soft paper towel (in one direction). Close the arm and retry measuring the sample.
	Dirty chamber	Wipe that was too wet or old	Wipe chamber with a fresh soft tissue lightly moistened with 70% ethanol.
27	Too low a cell concentration for seeding	Growth problem with culture	Repeat the assay with a fresh colony or fresh transformation.
		Lost cells during washing	Ensure that the cell pellet remains intact; carefully pipet off the supernatant.
45	Very high SEM, high variation between independent assays	Typo in data transfer or calculation error	Recheck the original data and Excel calculations, especially linked cells.
		Problem in one assay	Verify that all assay conditions are consistent (media, colony quality—plates should not be used for >1 month). Check if empty vector permeability is low. If not, there was a problem with the assay and it should be omitted from analysis. Perform an outlier test to identify abnormal variations. Repeat the assay to reduce variation. Rotate the position of samples in the incubator, as edges may experience variations in shaking speed or temperature.

### Statistical Analysis

For statistical analysis, refer to the detailed methods described in Basic Protocol part 8, Data evaluation, including the use of Boltzmann fitting, Grubbs’ outlier test, and SEM calculations for comparing constructs and conditions.

### Understanding Results

#### Yeast strains

In yeast, urea is transported into the cell via the DUR3 transporter, a member of the sodium solute symporter family, which co‐transports urea and protons. Because of its high polarity, urea exhibits low passive membrane permeability (Chen, 2013). The YNVW1 strain, which has a deletion of the *DUR3* gene (see Table 1), is widely used for urea complementation assays, as it lacks endogenous urea transport (ElBerry et al., 1993; Liu, Ludewig, Frommer, et al., 2003; Stoib, Shojaei, Siligan, et al., 2025; Stoib, Shojaei, Fischer, et al., 2025; Wang et al., 2012; Zanin et al., 2014).

Ammonia, another nitrogen source for yeast, is transported via the methylammonium/ammonium permeases (MEP1‐3). With a p*K*_a_ of 9.25, ammonia exists primarily as ammonium (NH_4_⁺) under physiological pH conditions. Distinguishing whether a candidate channel facilitates ammonia (NH_3_) or ammonium (NH_4_⁺) transport is challenging—especially if the channel itself is pH‐gated. For ammonia complementation assays, the 31019b Δ*mep1‐3* strain is used because it lacks endogenous ammonia transporters (Beitz et al., 2006; Ganz et al., 2020; Jahn et al., 2004; Kirscht et al., 2016; Loque et al., 2005; Stoib, Shojaei, Siligan, et al., 2025; Stoib, Shojaei, Fischer, et al., 2025).

#### Transformation

Competent yeast cells are prepared and transformed using the Yeast Trafo Kit 1 (Sigma), which employs the lithium acetate transformation method (Ito et al., 1983). Lithium ions chemically render yeast cells competent, and single‐stranded carrier DNA and polyethylene glycol (PEG) enhance plasmid uptake during a 42°C heat shock, increasing transformation efficiency (Kawai et al., 2010).

Both yeast strains (YNVW1 and 31019b Δ*mep1‐3*) carry a nonfunctional *URA3* gene. The pYES2 plasmid, which contains a functional *URA3* gene, serves as a selection marker. Only successfully transformed cells can grow on DOB‐ura selection plates. To maintain plasmid presence throughout the assay, uracil is omitted from all growth media.

#### Inoculation and induction

For inoculation, fresh colonies are selected from the selection plates and incubated overnight in DOB‐ura selection medium. The following day, cultures are diluted and grown to the logarithmic (exponential) phase, where cells are metabolically active and nutrient rich. To induce expression of the candidate protein, the *GAL1* promoter is activated by switching the carbon source from glucose to galactose. Residual glucose must be thoroughly washed away, as it represses the *GAL1* promoter. Galactose is used as the sole carbon source in the assay media to maintain continuous protein expression. Cultures are incubated for 24 hr to ensure that high levels of the candidate protein are present at the start of the urea/ammonia permeability assay.

#### Complementation assay principle

To assess urea and ammonia permeability, candidate channels are exposed to media containing only one nitrogen source. As nitrogen is essential for amino acid and nucleotide metabolism, cells unable to import nitrogen exhibit slowed or stagnant growth. The permeability of the candidate channel is evaluated by tracking cell growth and comparing it to both a negative control and a control medium.

The negative control consists of the deletion strain transformed with an empty vector backbone, which accounts for any effects of the transformation process or vector presence. Growth of the negative control reflects passive diffusion of the solute across the membrane and the baseline sensitivity of the deletion strain under the assay conditions, and it should remain consistently low.

To assess the overall fitness of the cells and the functionality of their nitrogen metabolism, cultures are also grown in arginine medium. Arginine is metabolized into urea and further into ammonia, but it is transported into the cell via CAN1 and GAP1, independent of the deleted endogenous transporters. This provides a positive control for cell viability and metabolic activity.

### Exemplary data

#### Urea uptake assay

This protocol describes a method for determining relative urea and ammonia permeabilities using yeast complementation assays. To demonstrate its application, we investigated the pH‐dependent gating of the bacterial proton‐gated urea channel *Hp*UreI from *Helicobacter pylori*. We measured the growth of the yeast deletion strain YNVW1 Δ*dur3*, transformed with either *Hp*UreI or an empty vector, across a pH range of 4.0 to 7.0 in a urea complementation assay. Cultures were incubated for 2 days in media containing urea as the sole nitrogen source.

Growth curves revealed distinct pH‐dependent patterns for *Hp*UreI (Fig. 2A). The day‐averaged cell counts were plotted against pH for both the negative control and WT *Hp*UreI (Fig. 2B). Cells expressing the empty vector showed minimal growth in urea media across all pH conditions, with growth observed only in arginine control medium. In contrast, cells expressing *Hp*UreI exhibited enhanced growth at acidic pH values (pH 4.0‐5.5), whereas growth diminished at higher pH levels (pH 6.5‐7.0). If the assay shows no difference in cell concentration between urea and arginine media, or if arginine growth is generally low, the assay should be repeated with fresh medium and newly transformed cells. For further analysis, cell counts were normalized to the arginine medium cell count, providing an internal standard to account for variations in cellular fitness. The relative urea permeability of each construct was plotted against the medium pH (Fig. 2C). The empty vector displayed consistently low permeability across the pH range (4.0‐7.0), whereas *Hp*UreI showed pH‐dependent permeability, with high urea uptake at acidic pH (4.0‐5.0) that decreased to background levels at neutral pH (6.5‐7.0).

To explore the functional impact of mutations designed to facilitate protein labeling for *in vitro* experiments, we compared the wild‐type (WT) *Hp*UreI to two mutants: A57C, located in periplasmic loop 1, and a construct with an N‐terminal tag (GPGSGS) combined with L134C, where L134C is located in periplasmic loop 2. Both mutations introduce cysteine residues for site‐specific labeling. The average relative urea permeability was calculated from multiple independent assays (WT *Hp*UreI, *n* = 18; A57C, *n* = 3; N‐tag + L134C, *n* = 5), with results plotted as mean ± SEM and fitted using a sigmoidal Boltzmann function (Fig. 2D). The p*K*a of WT *Hp*UreI, representing the pH at which half of the channels are open, was determined to be 5.69 ± 0.06. Both mutants (A57C: p*K*a 5.58 ± 0.15; N‐tag + L134C: p*K*a 5.58 ± 0.02) exhibited similar pH dependency to the WT, with slightly decreased p*K*a values and increased Δ_urea_ (Fig. 2E). The N‐terminal tag did not significantly alter pH gating or permeability compared to the single‐point mutation, aligning with previous findings (Stoib, Shojaei, Siligan, et al., 2025). These results suggest that the mutations induce only minor changes in urea permeability compared to WT *Hp*UreI.

#### Ammonia uptake assay

To demonstrate the versatility of complementation assays, we present data from an ammonia uptake assay comparing the known ammonia transporter *h*AQP8, the *Hp*UreI channel from *H. pylori*, and its pore‐lining mutant E177Q. The assay utilized the ammonia uptake‐deficient yeast strain *Saccharomyces cerevisiae* Sc18‐Δ*mep‐3*, transformed with plasmids encoding the respective channels. Cell growth was measured in media containing 2 mM ammonia as the sole nitrogen source, ensuring that only cells expressing functional ammonia‐permeable channels could proliferate. Growth was monitored over 48 hr across a pH range of 4.0‐7.0. Medium containing 1 mM arginine served as a control for overall cell fitness.

Growth curves for *h*AQP8 are shown in Figure 3A, with the average cell count on the second day plotted against pH conditions (Fig. 3B). The empty vector control exhibited minimal ammonia permeability across the entire pH range, with robust growth only in arginine‐containing control medium. In contrast, *h*AQP8 displayed significant ammonia permeability, which was higher at acidic pH and decreased at neutral pH, nearly matching the growth observed in arginine control medium.

To account for variations in cell fitness, cell counts were normalized to the arginine control growth, allowing calculation of relative ammonia permeability (Fig. 3C). This normalization highlighted the pH dependency of *h*AQP8's ammonia permeability. For final analysis, relative ammonia permeabilities from 3 to 18 independent measurements were averaged, and the standard error of the mean (SEM) was calculated. Data were fitted using sigmoidal or linear models (Fig. 3D).

WT *Hp*UreI also demonstrated pH‐dependent ammonia permeability, with higher flux at acidic pH and reduced flux at neutral pH. However, the E177Q mutant exhibited pH‐independent ammonia permeability at a lower relative level. The p*K*_a_ and amplitude of ammonia permeability were further characterized (Fig. 3E). hAQP8 had a p*K*_a_ of 6.19 ± 0.25, slightly higher than WT *Hp*UreI (5.90 ± 0.21), and a greater amplitude of relative ammonia permeability. Because E177Q lacked pH gating, no p*K*_a_ could be determined; only its relative permeability amplitude was reported. This mutation reduced permeability by approximately 50% and abolished pH gating.

This assay identified *h*AQP8 as a pH‐gated ammonia/ammonium channel, a property not previously described, as prior studies had focused solely on neutral pH conditions (Saparov et al., 2007; Soria et al., 2010). The pH‐dependent ammonia permeability of WT *Hp*UreI aligns with earlier findings (Stoib, Shojaei, Siligan, et al., 2025), whereas the pH‐independent behavior of the E177Q mutant underscores the assay's ability to distinguish between pH‐gated and pH‐independent constructs.

### Time Considerations

**Figure 6:**
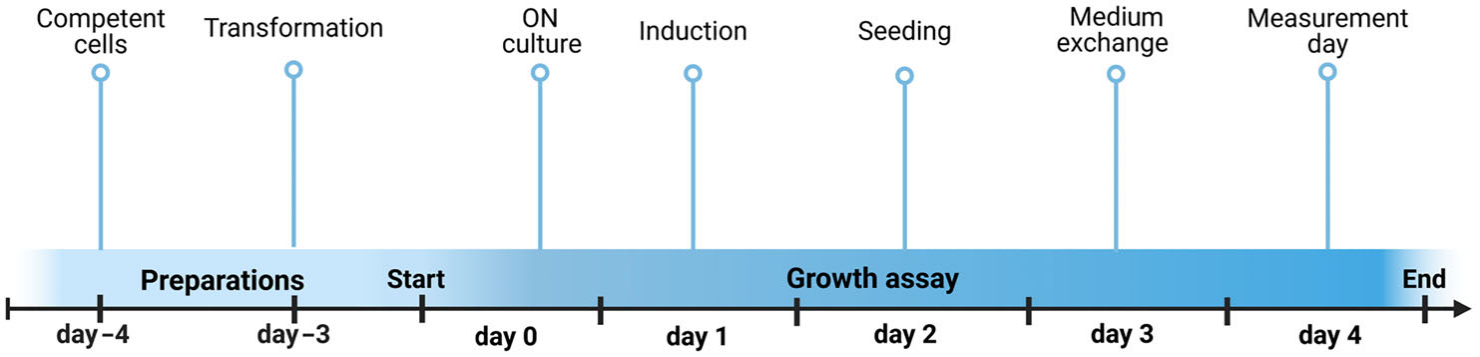
Timeline of growth assays. A schematic overview of the step‐by‐step timeline for conducting nitrogen complementation growth assays, including preparation, transformation, induction, seeding, medium exchange, cell growth measurement, and data evaluation.

An overview of the assay timeline is provided in Figure 6.

The estimated time required for each step is as follows:

- Part 1 (steps 1‐5), preparation of competent cells: 2 days
- Part 2 (steps 6‐14), plasmid transformation: 1.5 hr for preparation of four constructs + 2‐4 days for incubation
- Part 3 (steps 15‐16), Day 0—inoculation of cell cultures: ∼10 min
- Part 4 (steps 17‐25), Day 1—induction of protein expression: 5‐6 hr
- Part 5 (steps 26‐31), Day 2—seeding in urea pH media: 4 hr
- Part 6 (steps 32‐34), Day 3—medium exchange: 1 hr for four samples
- Part 7 (steps 35‐39), Day 4—measurement of cell growth: 7‐8 hr for four samples
- Part 8 (steps 40‐53), data evaluation: 1 hr

In our lab, four constructs are tested in parallel across eight conditions (32 samples total) in a single assay. By initiating three independent assays on consecutive days, up to 96 samples can be analyzed within 7 days.

### Author Contributions

A.H. conceived the project. C.S. and S.S. prepared the constructs. A.S. established and optimized the urea growth assay, and S.S. optimized the ammonia assay. A.S. evaluated the growth assays and created the artwork. A.S. and A.H. wrote the manuscript. All authors discussed the results, provided feedback on the manuscript, and approved the final version.

### Conflict of Interest

The authors declare no conflict of interest.

## Data Availability

The data supporting the findings of this study are openly available in zenodo at https://zenodo.org/uploads/17975811, reference number 10.5281/zenodo.17975811.
